# Physical but not virtual presence of others potentiates implicit and explicit learning

**DOI:** 10.1038/s41598-022-25273-4

**Published:** 2022-12-08

**Authors:** Pietro Sarasso, Irene Ronga, Elena Del Fante, Paolo Barbieri, Irene Lozzi, Nicola Rosaia, Alessandro Cicerale, Marco Neppi-Modona, Katiuscia Sacco

**Affiliations:** 1 BIP (BraIn Plasticity and Behaviour Changes) Research Group, Department of Psychology, University of Turin, Via Verdi, 10, 10124 Turin, Italy; 2 Department of Economics, Harvard University, Cambridge, MA USA

**Keywords:** Neuroscience, Psychology

## Abstract

E-learning activities are becoming more and more common. Whilst it is well known that the physical presence of others motivates individuals to engage in perceptual and learning tasks, systematic investigations comparing the effects of physical and virtual co-presence of others on knowledge acquisition are still scarce. Here we investigate the effects of physical and virtual co-presence of others on explicit and implicit learning. In Experiment 1 (discovery sample), retrieval accuracy in a spatial memory task and EEG indexes (mismatch negativity-MMN) of implicit perceptual learning were recorded when participants were alone or in presence of another individual. In Experiment 2 (replicating sample), we added a “virtual” condition, where the same tasks were performed during a video-conference call. In both experiments, MMN was demonstrated to encode for perceptual learning as revealed by the significant correlation with Bayesian Surprise (a consolidated information-theoretic index of Bayesian learning). Furthermore, In Experiments 1 and 2 physical co-presence systematically ameliorated memorization performances and increased MMN indexes related to implicit learning. These positive effects were absent in the virtual condition, thus suggesting that only physical, but not virtual co-presence is effective in potentiating learning dynamics.

## Introduction

Although the use of on-line education offers significant practical, economic and safety (as during the Covid-19 pandemic) advantages to both students and teachers, the effects of physical vs. virtual presence of others (i.e., peers and teachers) on learning outcomes is still poorly investigated. A better understanding of the differences and similarities between ‘in presence’ and online learning is urgently needed to orient present and future decisions of teachers, institutions and policymakers regarding the use of online teaching activities.

Sharing information with others is vital for collective coordination^1^ and we might have evolved to prefer information that is shared^2^. Indeed, social psychology revealed that sharing mental states with others^3^ favors the encoding of novel information, emotions and sensations^4^, because sensory outcomes of shared *vs.* solo experiences are amplified^5–7^, so that people devote greater cognitive resources to co-attended stimuli^8,9^ which undergo deeper processing^10,11^. In general, co-presence positively affects cognitive functions, such as attention to a target stimulus^12,13^, social and behavioural learning^9^, language acquisition^14^, memory^15–18^, intrinsic motivation^19^, dissonance reduction^20^ and behavioural control^21^. Overall, these findings are generally referred to as shared attention effects^17^. To date, however, no experimental evidence of the electrophysiological correlates of such effects has been provided yet^22^.

Attentional resources are particularly likely to be directed to information that is co-attended with significant others, such as in-group members^13,15,18^, partners^23^ and caregivers^1,24^. Indeed, “psychological closeness”^23^ between co-experiencers moderates the amplification of shared experiences. Boothby et al.^6^, modulated psychological closeness by manipulating social (strangers vs. friends) and physical (being in the same room vs. being connected via live video feed) distance and found that only socially and physically closer co-experiencer showed amplified experiences. These results suggest that stimuli that are remotely co-experienced may not attract human attention as when shared with physically present co-attendants.

Other studies, however, suggest that virtual co-presence can mimic shared attention effects. Shteynberg et al.^9^ falsely told their participants that another participant was participating online, and they demonstrated that experience that is shared with a sham similar virtual other (who appeared to have chosen the same colour avatar as the participant) can foster social imitation, emotional intensification^8^ and the recall of a list of co-attended words^18^. Similarly, it has been recently shown that adult online learning of factual information is better for live interactive presentations than recorded video presentations^25^. These studies, however, were focused on the in-group/out-group variable or other social factors and did not directly compare physical and online co-presence conditions and the magnitude of the corresponding effects.

In the present study, we investigated whether (1) the physical presence of others, relative to their absence, potentiates memory, as well as implicit learning of sensory regularities; (2) the electrophysiological investigation of perceptual learning dynamics may uncover the underlying neural mechanisms of shared attention effects; and (3) the virtual presence of others (e.g. during a video-call) has a comparable effect to physical presence on memorization and perceptual learning.

**Figure 1:**
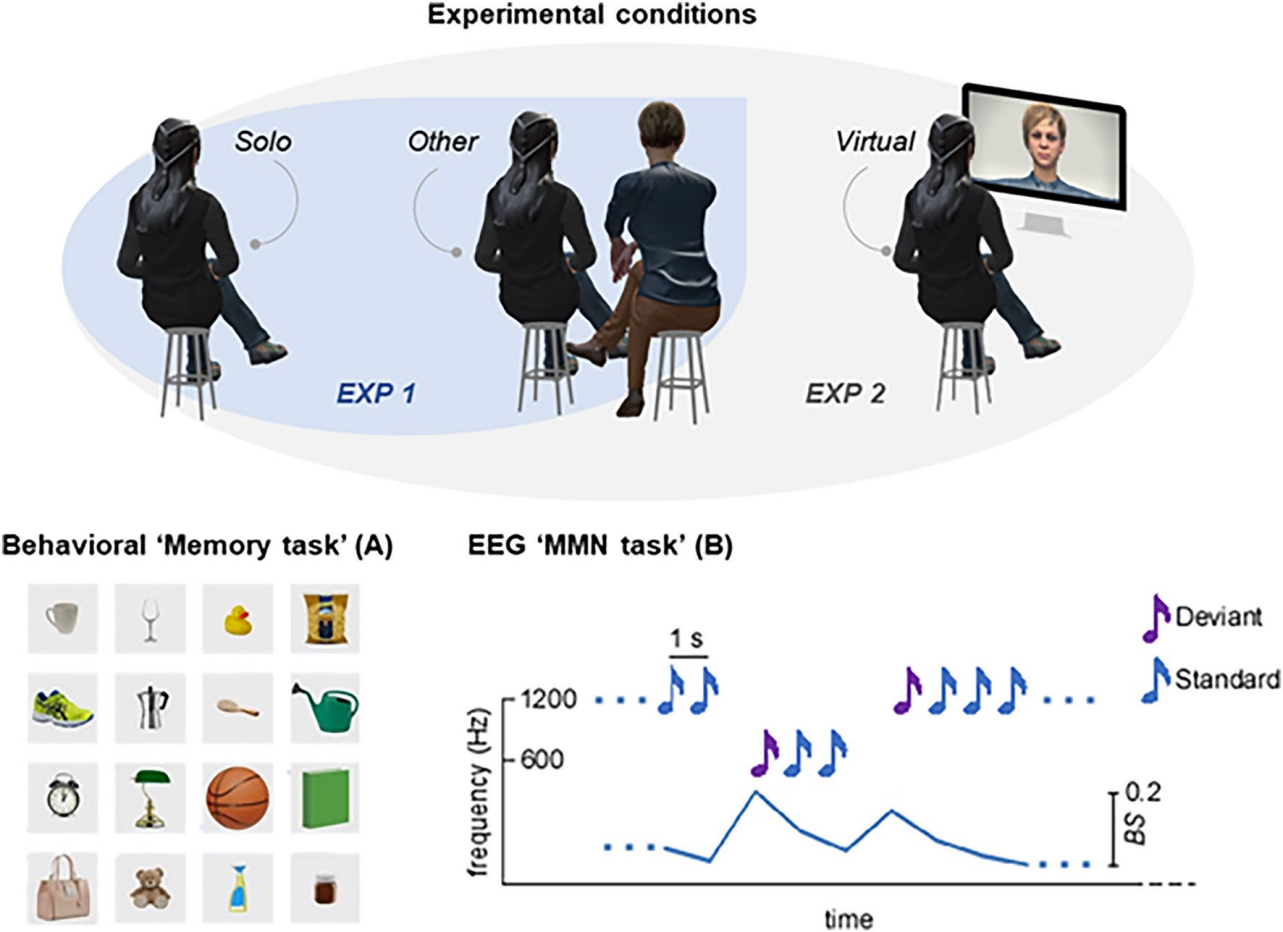
The top panel represents the experimental set-up across conditions in the two experiments. In Experiment 2, the screen used for the video-call was shown to the participant just before each experiment and subsequently placed next to the participant were the confederate sat in the *Other* condition. The screen is here represented in front of the participant for display reasons only. The screen was shown to the participant for a few seconds and then positioned next to the participants in the same position as in the *Other* condition (see “Stimuli and experimental design”). The left bottom panel represents the pictures employed in the memorization phase of the memory task. The right bottom panel represents Standard and Deviant auditory stimuli employed in the MMN roving paradigm. Human figures were created with the Adobe software Mixamo (https://www.mixamo.com).

In Experiment 1 (discovery sample), participants (N = 18) performed a spatial memorization task (Experiment 1a) and an EEG task (1b) in two different ‘presence’ conditions: *Solo* (alone) and *Other* (together with a confederate—see Fig. 1). In Experiments 2a–b (replicating sample), we replicated the same tasks, with the inclusion of an additional *Virtual* condition (the confederate participated via video-call). Behavioural Exp. 1a–2a consisted of a traditional spatial memory task (similar to the popular “memory” game), where participants were asked to memorize and then recall the spatial locations of common objects. In EEG Exp. 1b–2b, participants were asked to listen to a stream of higher or lower frequency (Hz) sounds, while we registered their mismatch negativity (MMN) response, a differential wave obtained by subtracting the neural response to Standard events from that of Deviant events^26^. The MMN response to frequency—in Hz—deviant sounds, peaking between 150 and 250 ms post-stimulus onset, is thought to result from the activations of the posterior auditory cortex (superior temporal gyrus) and the inferior frontal gyrus^27,28^, and is often interpreted as a neurophysiological signature of implicit perceptual learning, i.e. indexing the update of the predictive models of the sensory environment^29–31^. Therefore, to verify such an association between MMN and perceptual learning in the present paradigm, in both EEG experiments we performed a point-by-point correlation between single-trial amplitude fluctuations and *Bayesian Surprise*, a well-known theoretic index of perceptual learning^30,32^ (see “Methods” and SI).

Coherently with previous behavioural studies, we expected to observe improved explicit and implicit learning in the *Other* as compared to the *Solo* condition, expressed by better memorization performances (Exp. 1a) and larger MMN responses (Exp. 1b), respectively. Moreover, if virtual presence similarly influences memorization as physical presence, in Experiments 2 we expect to observe comparable results in *Other* and *Virtual* conditions.

## Methods

### Experiment 1 (discovery sample)

#### Participants

Eighteen healthy right-handed subjects participated in Experiment 1 (13 females; mean age: 26.95 years, ± 2.98 SD; mean years of education: 18.12, ± 1.75 SD). Participants were homogeneous in terms of education, most were graduate university students. All participants gave their written informed consent to participate in the study, which conformed to the standards required by the Declaration of Helsinki and was approved by the Ethics Committee of the University of Turin (Prot. n. 121724—01/03/18). Participants were not compensated for taking part in the experiment.

Sample size (N = 18) was a priori determined through a power analysis based on the effect size obtained in a pilot experiment identical to the main behavioural experiment, involving 6 additional participants and exploring spatial memorization accuracies (ACC) between *Other* and *Solo* conditions (Cohen’s d = 0.823; α = 0.05; required power = 0.95). Additionally, since there is not an established method for estimating the required sample of properly powered point-by-point cluster-based statistics (see “Data analysis”) in EEG studies, we followed Brysbaert's guidelines^33^ which suggest that when power is unknown one should to assume a medium effect sizes (d = 0.5), and run the required amount of participants according to the specific design, which in our case is a repeated measure test of a main effect (see Table 7 in Brysbaert^33^; required sample = 18; required power = 0.8). The resulting required sample was similar to the ones in previous studies where we employed the same EEG analyses^34–36^. Moreover, to maximize statistical power and reduce the probability of false positives^37^ we planned the collection of two independent discovery and replicating samples (see “Experiment 2 (replicating sample)”).

#### Stimuli and experimental design

The experiment was based on a within-subject design. Each participant performed the experiment in two experimental conditions. The experiment (Fig. 1) consisted of two sessions, each divided into two different conditions (i.e., *Solo* and *Other*). In the *Solo* condition participants were alone in the experimental room during the tasks. In the *Other* condition, subjects sat next to another individual. For each condition participants had to perform two different tasks: a behavioural ecological task (i.e. ‘memory task’) and an electrophysiological task (i.e. ‘EEG Mismatch Negativity task’). The two experimental tasks were performed in two different days. The order of presentation of both conditions and tasks was randomized across subjects.

To test explicit learning and memorization, in the *memory task* we employed the *memory V.R. test*, an object-location memory test developed in collaboration with SynArea Consultants Srl (Turin, Italy). Object location tests have been proved to be an effective assessment for explicit memory competences^38^. The task was developed using Unity software (https://unity3d.com). The visual stimuli employed were 16 everyday-life object images (i.e., ball, glass, book) presented in a 4 rows × 4 columns pattern. The *memory V.R. test* consisted in two phases, one devoted to memorization and the other to recall (see “Memory task”). Importantly, in the *Other* condition, the confederate sat next to 4 participants only in the memorization phase.

The *EEG MMN task* was designed to investigate implicit perceptual learning. Converging evidence from electrophysiological studies suggests that the MMN elicited by the presentation of sounds that deviate from a pattern established by the preceding inputs^39,40^, is typically considered a neurobiological marker of implicit perceptual learning of sensory regularities^41,42^. If co-presence of another subject modulates implicit perceptual learning, we expect to observe significantly different MMNs in the two scenarios.

EEG was registered while subjects listened to sound sequences. Deviant and Standard sounds were presented according to the classic roving paradigm with two sounds differing in their frequency (Hz; high-pitch and low-pitch). For further details refer to the SI.

During the experiments, participants sat at a table with eyes open, in front of a 53 cm (21 inches) computer screen. The screen centre was aligned with the subjects’ trunk midline. Each arm of the participant arms was resting on the corresponding leg during the MMN roving paradigm*.* In the *Other* condition, the confederate sat on a chair one meter away from the participant, so that they could easily see the computer screen and hear the sounds played via the loudspeakers. Both participants and confederates were asked to remain silent and fixate the computer screen during the experiments.

#### Memory task

Subjects performed the *memory task* twice, once for each condition. Each of the two condition had a total duration of 27 min. Each experimental block started with the memorization phase (duration: 2 min), where participants were asked to pay attention to the location of the objects (see Fig. 1) and try to memorize it. Subsequently, subjects performed a distraction task for 5 min, to avoid any rehearsal effects. In the final recall phase, participants were asked to place the 16 objects in the exact position displayed in the memorization phase. Each object placement had to be completed within 10 s, and was tracked by a green bar displayed on the screen under the object. The experimental block (memorization phase, distraction task and recall phase) was repeated three times for each condition (i.e., *Solo* and Other), each block presenting a different random-generated objects layout. In the *Other* condition, participants were told they were going to memorize locations together with their confederate, but for logistic reasons both of them were going to recall them in separate rooms. We specified that it was not a competition and that their data were not going to be compared. The presentation software automatically registered reaction times and response accuracy.

#### EEG MMN task

Here we investigated whether and how implicit perceptual learning is modulated by contextual factors, such as the presence of another person. To test this hypothesis, in each of the two sessions, one for each condition, participants performed 2 runs of a standard roving paradigm while we registered their EEG activity. Sound sequences consisted of trains of 1152 stimuli per run. During the *Other* condition, participants and confederates were simply asked to remain silent, look straight ahead at a central fixation cross on the screen and listen to the sound sequences; during the *Solo* condition, participants performed the same *EEG MMN task* alone. The order of presentation of the two runs (total duration: approximately 40 min) was randomized across participants, as to exclude any specific sequence effect. Differently from traditional oddball paradigms^43^, where the repeated presentation of standard sounds is occasionally interrupted by the occurrence of physically different deviant sounds, in roving paradigms^44,45^ different stimuli (high-pitch and low-pitch intervals in our case) can represent both *Deviant* and *Standard* stimuli (Fig. 1).

High-pitch and low-pitch intervals were presented in consecutive trains of alternating pitch with a constant inter-stimulus interval of 1 s (in accordance with previous studies employing similar inter-trials^30^). Any time a change in the stimulation stream occurs (i.e., the passage from a high-pitch to a low-pitch stimulus train or vice versa), the first stimulus of the new train constitutes a *Deviant* event, since it differs from the preceding train of stimuli, which are therefore considered *Standard*^46^. The length of the trains of high-pitch and low-pitch intervals was chosen according to a pseudo-random order, so that both the number of presentations and the average value of the Bayesian surprise (see “Data analysis”) were equal across pitch types (i.e. high or low; Fig. 1). Moreover, the ratio between *Standard* (80%), and *Deviant* (20%) trials was kept constant across runs. Differently from traditional oddball paradigms, in roving protocols each stimulus type has exactly the same probability of occurrence, thus allowing to dissociate genuine effects of Bayesian perceptual learning from rarity-driven modulations.

#### Data analysis

##### Behavioral analyses

Single subjects’ (N = 18) mean accuracies corresponding to the *Solo* and *Other* condition from the *memory task* were compared with a paired sample two-tailed t-test. Behavioural results were corrected for multiple comparisons (total number of tests = 4; see Exp. 2a) by a Benjamini–Hochberg procedure with a false discovery rate of 5%.

#### Bayesian perceptual surprise computation

For a detailed description of the mathematical computations please refer to the Supplementary Information. Similarly to previous studies^30,32,35,47^, to relate single-trial EEG signals to Bayesian perceptual learning, we computed Bayesian surprise values for each single trial using a sequential Bayesian learning algorithm of stimulus probabilities, thus obtaining 1152 estimated surprise values (i.e., the total number of sounds composing the sequence presented in each session of the EEG MMN task).

#### Statistical analyses

ERPs belonging to the same condition (i.e., *Solo* or *Other*) and to the same type (i.e., standard vs. deviant) were then averaged, to obtain four average waveforms for each subject (i.e., *Solo Standard*, *Solo Deviant*, *Other Standard*, *Other Deviant*). For preprocessing details please refer to SI.

In the present study we employed point-by-point statistical tests. It is worth noting that point-by-point analyses represent a statistical approach common in EEG studies^48–52^, directed to highlight possible differences between conditions across the whole epoch time-course, without any a-priori assumption. One statistical comparison for each time point composing a waveform was performed. In order to correct for multiple comparisons, a cluster-based permutation testing approach (1000 random permutations; alpha level = 0.05; percentile of mean cluster sum = 95; minimum number of adjacent channels = 2) was employed to each point-by-point analysis^53^. Significant clusters were based on both temporal contiguity and spatial adjacency of a minimum of two electrodes. The so obtained clusters of significance represent the result of the point-by-point analyses, corrected by permutation testing. Hence, pre-processed epochs and Bayesian surprise values corresponding to single trials served as input for a point-by-point trial-by-trial correlation analysis^49,54^. For each participant and for each condition separately, the analysis computed the correlation between Bayesian surprise and trial-by-trial (N = 1152) fluctuations of the EEG signal registered at single channels. The outcome of the correlation analysis was two 1 s long time series of r-values for each channel and for each subject (from 0.15 s pre-onset to 0.85 s post-onset). This constituted the input for two group-level two-tailed point-by-point t-tests with permutation-based correction for multiple comparisons (1000 permutations; alpha level = 0.05; percentile of mean cluster sum = 95; minimum number of adjacent channels = 2). For the *Solo* condition and the *Other* condition separately, the test compared single subjects’ correlation coefficients against the constant 0 at each time point. This allowed us to identify time-clusters containing signal amplitudes which significantly correlated with Bayesian surprise indexing model update. Supposedly, these clusters contain waveforms that are significantly modulated by Bayesian perceptual learning. We expected to find greater correlation values around the time interval of the MMN, N2, P300, N4 indexes, in correspondence of the centro-parietal electrodes.

Subsequently, to explore the presence of a significant modulatory effect of co-presence on the MMN index, we computed MMN differential waves for each subject and for the two conditions separately. MMN responses were obtained by subtracting the ERP elicited by standard intervals (in this analysis we included only the last standard trial for each stimuli train occurring before deviant trials^30^; N = 208) from that elicited by deviant intervals^26^. Single participants’ MMN registered on single channels were then entered in group-level analyses. Since we were interested in testing for possible differences in MMN responses corresponding to the two conditions, we performed a point-by-point t-test^49^, with clustersize-based permutation correction for multiple comparisons based on temporal consecutivity (1000 permutations; alpha level = 0.05; percentile of mean cluster sum = 95; minimum number of adjacent channels = 2), on differential MMN (Deviant-Standard) values. The test compared single subjects’ MMN amplitudes corresponding to the *Solo* and *Other* conditions at each time point, for each channel separately. This allowed us to identify time-clusters containing MMN indexes of implicit perceptual learning which significantly differed between *Solo* and *Other* conditions.

### Experiment 2 (replicating sample)

#### Participants

Eighteen healthy right-handed subjects participated in the experiment (9 women, age mean = 26.22; ± SD =  ± 1.55; years of education, mean: 17.73; ± SD: ± 1.27). All participants gave their written informed consent to participate in the study, which conformed to the standards required by the Declaration of Helsinki and was approved by the Ethics Committee of the University of Turin (Prot. n. 121724—01/03/18). Participants were not compensated for taking part in the experiment.

#### Stimuli and experimental design

All procedures were identical to those employed in *Experiment 1*, except for the addition of a third condition, i.e., the *Virtual* condition, both in the behavioural and in the electrophysiological task. In this *Virtual* condition, the same confederate of the *Other* condition performed the memorization phase of the *memory task* and listened to the sound sequences together with the subject via a video-call (i.e. using Skype). To avoid confounding effects by keeping the different conditions (*Other* and *Virtual*) perceptually identical, after an initial phase where subjects were shown their partners on the computer screen (video-calls were started on a lap-top while the experiment was run on a different computer), the screen was placed next to the participant, in the same position where the confederate was in the *Other* condition. Participants were told that their confederate could see and hear the presented stimuli and, similarly to the *Other* condition*,* that confederates were going to perform the recall phase of the *memory task* separately for logistic reasons. Because time spent together and shared experiences can influence the psychological distance between participants and confederate, which in turn can amplify shared attention effects^6^, procedure were kept as similar as possible between the *Other* and *Virtual* conditions: in both conditions participants and confederates were asked to look at each other for a few seconds and say hello to each other. After this minimal familiarization, (which was conceived mainly to exclude the possibility that participants believed that the videocall was registered and no one was taking part to the experiment together with them) participants were asked to look straight ahead at the fixation cross. Similarly to *Experiment 1*, both the participant and the confederate were asked to remain silent throughout the experiments.

#### Data analysis

Statistical analyses were identical to those employed in *Experiment 1*, with the only exception that the point-by-point correlation analysis was performed three times, once for each condition (i.e. *Solo, Other; Virtual*). Coherently, three point-by-point t-tests confronted MMNs values corresponding to three conditions (*Other* vs. *Solo*, *Other* vs. *Virtual*, *Virtual* vs. *Solo*).

The datasets analysed (Single subjects’ results from Experiment 1 and 2) during the current study and the experiment code generated by the e-prime presentation software are available in the Mendeley repository [Sarasso, Pietro (2022), “Whith or without you—MMN in virtual and physical co-presence”, Mendeley Data, V2, https://doi.org/10.17632/9sj977x4j4.2]. Neither of these experiments was formally preregistered.

## Results

### Experiment 1

#### Spatial memory task

**Figure 2:**
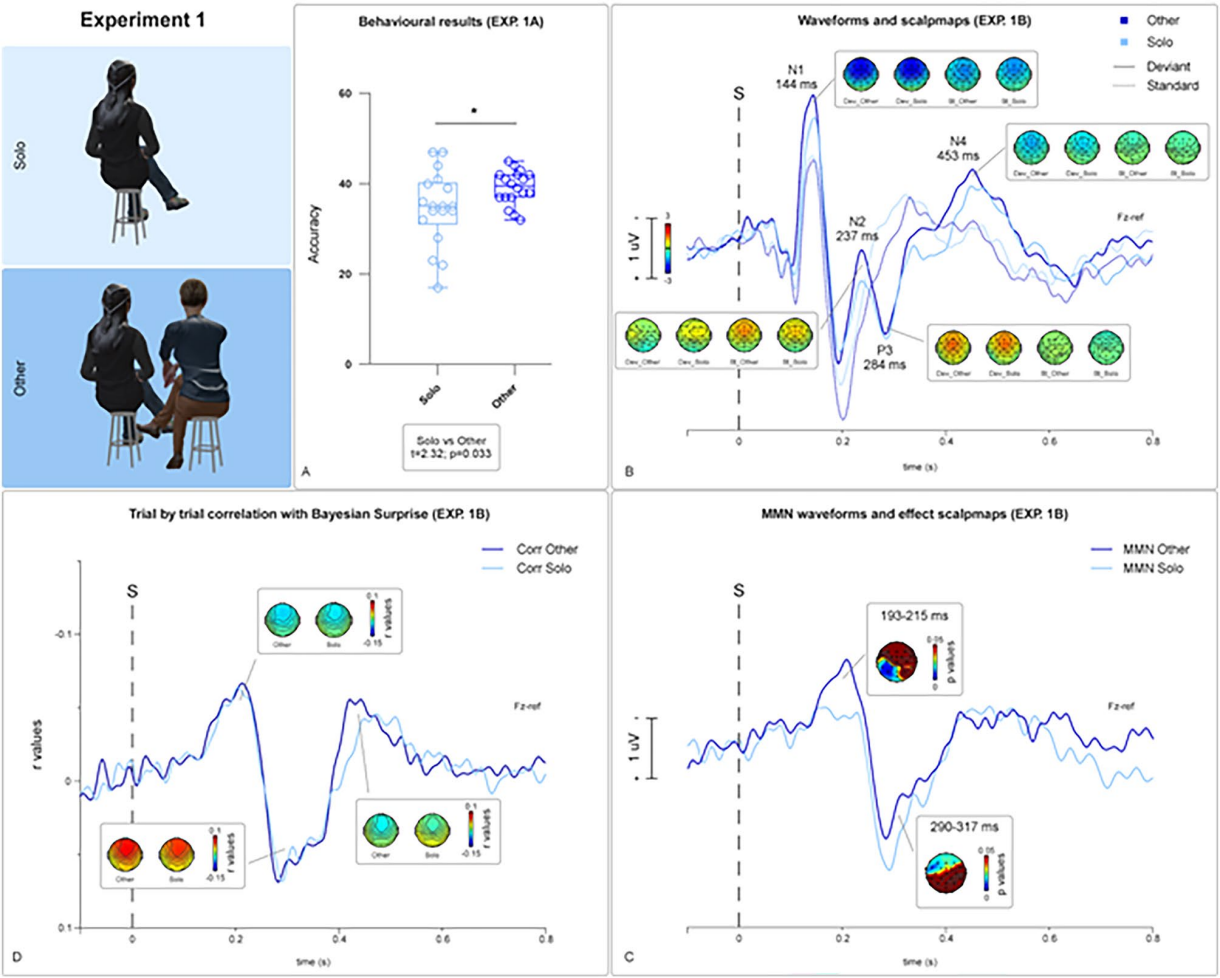
Whisker Plots in (**A**) depict behavioural results from Experiment 1a corresponding to the *Solo* (light blue) and *Other* (blue) conditions. Dots represent single subjects’ mean accuracies. Panel (**B**) shows average waveforms and peak amplitudes distribution on the scalp for relevant components (N1, N2, P3, N4) from Experiment 1b. Dotted and solid lines represent average ERPs corresponding to Standard and Deviant trials respectively. MMN differential waveforms are displayed in (**C**) (bottom-right) and show a negative peak at approximately 210 ms post-onset. Scalpmaps represent p values distribution across the scalp from the point-by-point t-test. Scalpmaps show results from the t-test at 200 and 300 ms corresponding to the effect peak latency of the two significant time clusters (193–215 ms and 290–317 ms) surviving permutation testing. Panel (**D**) (left-bottom) represents average r values from the point-by-point correlation analysis. Correlation between Bayesian Surprise and trial-by-trial amplitudes peaked at 210 ms post onset, corresponding to the MMN peak latency. Human figures in panel A were created with the Adobe software Mixamo (https://www.mixamo.com).

The effect of physical co-presence was evaluated by comparing memorization accuracies in the *Solo* condition (*M* = 34.62 (72.125%); *SD* = 8.2) with accuracies from the *Other* condition (*M* = 39.17 (81.25%); *SD* = 3.73). A paired sample t-test has been used for this purpose. Significant improvements were observed in memorization performances corresponding to the *Other* condition compared to the *Solo* condition, [*t*(17) = − 2.32, *p* = 0.033, *dz* = 0.58; Fig. 2a]. The present *p* value survived Benjamini–Hochberg correction for multiple comparisons (total number of performed tests = 4; false discovery rate 5%).

#### Trial-by-trial correlation with Bayesian surprise

The correlation analysis between single trial amplitudes and Bayesian Surprise indicated that r values peaked over frontal electrodes (Fz) at similar latencies for *Other* and *Solo* conditions (Fig. 2d). At Fz, r values corresponding to the *Other* and *Solo* both significantly differed from the costant 0 in three significant clusters which survived cluster correction, corresponding to the latencies of the MMN, the N2-P3a complex and the N4 components of the auditory evoked potential. In the following lines we report the latency of significant clusters at Fz for the *Other* condition (parentheses indicate p values and t values at peak latencies): 130–244 ms (*p* < 0.001; *t* = − 6.39); 262–375 ms (*p* < 0.001; *t* = 5.67); 499–497 ms (*p* < 0.001; *t* = − 6.43). In the following lines we report the latency of significant clusters at Fz for the *Solo* condition: 143–240 ms (*p* < 0.001; *t* = − 6.1); 265–383 ms (*p* < 0.001; *t* = 6.04); 418–551 ms (*p* < 0.001; *t* = − 4.4). These results confirmed our prediction, indicating that MMN (peaking at 210 ms post-onset) indexes Bayesian perceptual learning in our study. Furthermore, and in accordance with data from the literature, we also found a correlation between model updating following surprising stimuli (i.e. sensory surprise) and the P3 and N400 components^55,56^.

#### MMN results

ERPs elicited by the *Other* and *Solo* conditions on Fz (where MMN peak amplitudes are registered) are reported in Fig. 2b. Grand-average waveforms were comparable with previous studies on auditory frequency processing^40,57^. For both *Other* and *Solo* conditions, the MMN differential wave obtained by subtracting Standard from Deviant average response, showed a negative peak over frontocentral at approximately 210 ms postonset, coherently with previous findings^40^ (Fig. 2c). Crucially, the point-bypoint t-test performed on MMN differential waves (*Other* vs. *Solo*) revealed two significant time-clusters surviving cluster correction. The first centro-parietal cluster spanned across C3, Cp5, Cp1 and P7 electrodes at 193–215 ms post-onset (Fig. 2c), which corresponds to the MMN peak latency. This result confirmed that, as expected, MMN waveforms were significantly larger in the *Other* condition compared to the *Solo* condition. The scalp location of the effect of condition on MMN differential waves is coherent with previous studies that localized the source of MMN responses to frequency deviance in the superior temporal gyrus^27,28^. Müller et al. PET study^58^ showed that when contrasting frequency deviant and frequency standard sounds the largest magnitude of activation to frequency deviant sounds emerged in the lower part of the left posterior superior temporal gyrus. The second cluster surviving cluster correction is centred over frontal electrodes (Fp1, Fpz, Fp2, Fz, F4, Fc5) at 290–317 ms post-onset.

As it can be visualised in Fig. 2b this is possibly due to a more negative rebound of the P2 component following the presentation of Standard trials in the *Solo* condition (following a larger P2 component in the *Other* condition).

### Experiment 2

#### Behavioral memory task

**Figure 3:**
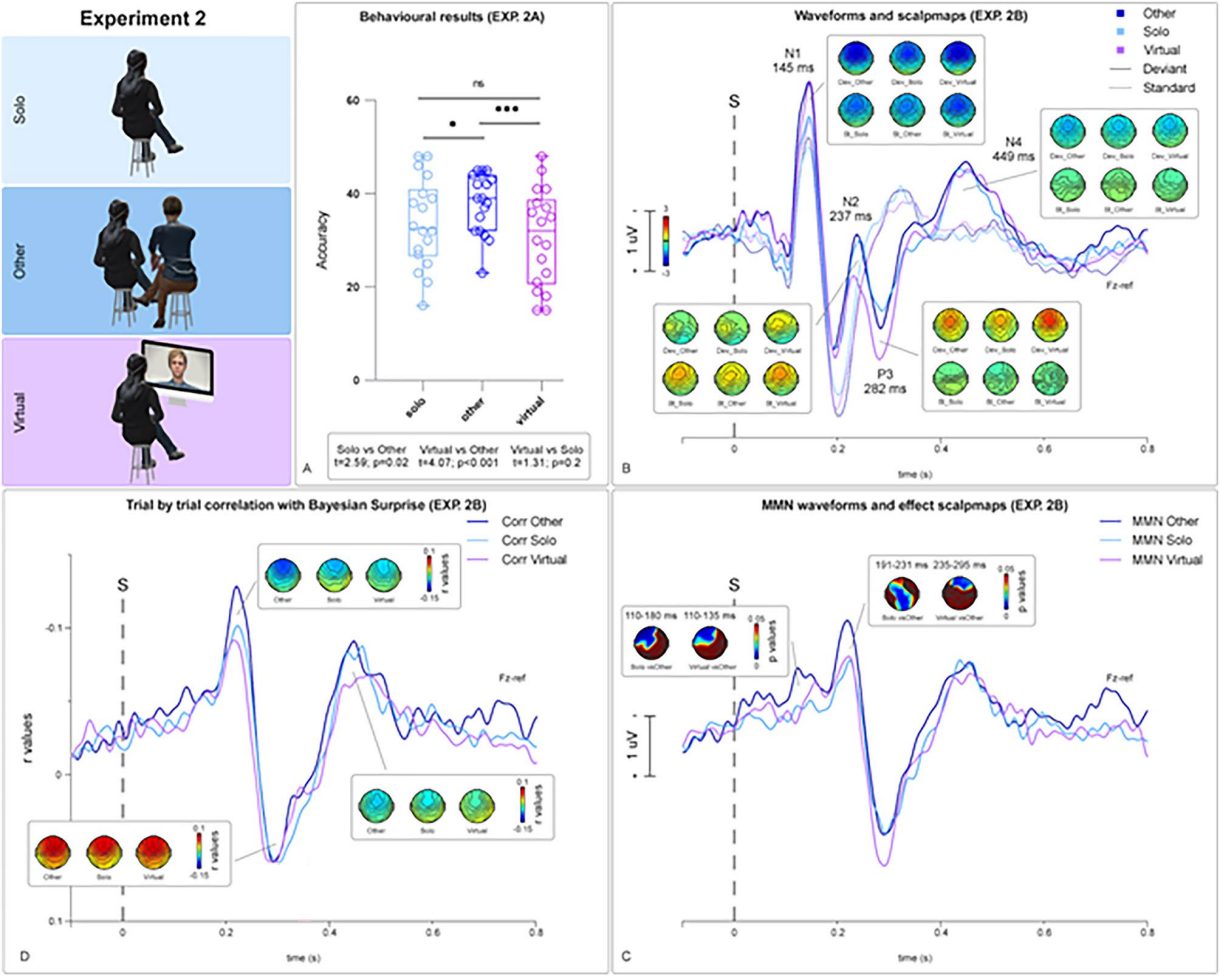
Whisker Plots in (**A**) depict behavioural results from Experiment 2a corresponding to the *Solo* (light blue), *Virtual* (purple) and *Other* (blue) conditions. Dots represent single subjects’ mean accuracies. Panel (**B**) shows average waveforms and peak amplitudes distribution on the scalp for relevant components (N1, N2, P3, N4) from Experiment 2b. Dotted and solid lines represent average ERPs corresponding to Standard and Deviant trials respectively. MMN differential waveforms are displayed in (**C**) (bottom-right) and show a negative peak at approximately 210 ms post-onset. Scalpmaps represent p values from point-by-point t-tests (*Solo* vs. *Other*; *Virtual* vs. *Other*), corresponding to the effect peak latency of significant time clusters (*Solo* vs. *Other*: 110–180 ms and 191–231 ms; *Virtual* vs. *Other*: 110–135 ms and 235–295 ms) surviving permutation testing. Panel (**D**) (left-bottom) represents average r values from the point-by-point correlation analysis. Correlation between Bayesian Surprise and trial-by-trial amplitudes peaked at 210 ms post onset, corresponding to the MMN peak latency. Human figures in panel A were created with the Adobe software Mixamo (https://www.mixamo.com).

Mean accuracies (Fig. 3a) peaked in the *Other* condition [*M* = 38.11 (79.4%); *SD* = 6.4] and were reduced in the *Solo* condition [*M* = 33.72 (70.25%); *SD* = 9.47] and in the and *Virtual* condition [*M* = 30.61 (63.77%); *SD* = 10.39].

The effect of physical and virtual co-presence was further evaluated by comparing memorization accuracies corresponding to the three different conditions by means of three two-tailed paired sample t-tests (*Other* vs. *Solo*; *Other* vs. *Virtual*; *Virtual* vs. *Solo*). Significant enhancements in memorization performances were observed in the *Other* condition compared to the *Solo* condition [*t*(17) = 2.59; *p* = 0.019, *dz* = 0.73] and in the *Other* condition compared to *Virtual* condition [*t*(17) = 4.07; *p* < 0.001, *dz* = 1.06]. Memorization performances in the *Virtual* and *Solo* conditions were not significantly different [*t*(17) = 1.31; *p* = 0.207; *dz* = 0.31]. All significant *p* values survived Benjamini–Hochberg correction for multiple comparisons (total number of performed tests = 4; false discovery rate 5%).

#### Trial-by-trial correlation with Bayesian surprise

As in Experiment 1, the trial-by-trial correlation analysis between single trial amplitudes and Bayesian Surprise indicated that r values peaked over fronto-central electrodes at three different latencies, similarly for the *Other*, *Solo* and *Virtual* conditions (Fig. 3d). Significant clusters corresponded to the latencies of the MMN, the N2-P3a complex and the N4 components of the auditory evoked potential. At Fz, results from the trial-by-trial correlation analysis performed on EEG responses following the presentation of the three conditions, *Other*, *Solo* and *Virtual* intervals evidenced three significant clusters (i.e. r values significantly differed from the constant 0). For the *Other* condition: 59–249 ms (*p* < 0.001; *t* = − 9.27); 264–347 ms (*p* < 0.001; *t* = 4.88); 405–522 ms (*p* < 0.001; *t* = − 5.84). For the *Solo* condition: 129–247 ms (*p* < 0.001; *t* = − 6.71); 265–362 ms (*p* < 0.001; *t* = 5.66); 407–497 ms (*p* < 0.001; *t* = − 5.78). For the *Virtual* condition: 129–236 ms (*p* < 0.001; *t* = − 5.72); 257–336 ms (*p* < 0.001; *t* = 8.52); 419–555 ms (*p* < 0.001; *t* = − 4.18). These results confirmed our prediction, indicating that MMN indexes Bayesian perceptual learning in our study. Furthermore, and in accordance with data from the literature, we also found a correlation between model updating following surprising stimuli and the P3 and N400 components^55,56^.

#### MMN results

ERPs elicited during the *Other*, *Solo* and *Virtual* conditions intervals over Fz are reported in Fig. 3b. Grand-average waveforms were similar to Experiment 1 and comparable with previous studies on auditory frequency processing^40^. For *Other*, *Solo* and *Virtual* intervals, MMN waveforms showed a negative peak over fronto-central electrodes at approximately 220 ms post-onset (Fig. 3c). The point-by-point t-test comparing MMN waveforms registered over Fz during the *Other* and *Solo* conditions (*Other* vs *Solo*) revealed two early significant clusters corresponding to the N1 and MMN latencies, and a later significant frontal cluster at approximately 700 ms post onset. The first smaller cluster, corresponding to the N1 negative peak spanned across frontal electrodes (Fp1, Fpz, F7, F3, Fc5) at 110–177 ms post-onset. The second cluster including the time interval between 191 and 231 ms extending over a broad set of frontal, central and parietal electrodes (Fp1, Fpz, Fp2, F7, F3, Fz, FC5, FC1, FC2, FCz, T7, C3, Cz, CP1,CP2, CP6, P3, Pz, P4, Oz, O2 and POz) corresponded to the MMN peak latency. The point by point t-test comparing MMN registered during the *Other* and *Virtual* conditions revealed a single significant cluster including the time interval between 235 and 295 ms post-onset, corresponding to the descending portion of the MMN negative peak. The significant cluster spanned across frontal channels. More specifically, the results were significant over: FP1, Fpz, F3, Fz, F4, F8.

The t-test comparing *Solo* and *Virtual* conditions (*Solo* vs. *Virtual*) was not statistically significant. Overall, as expected, MMN waveforms were significantly larger in the *Other* condition (Fig. 3c).

## Discussion

In Experiment 1 (discovery sample), we demonstrated that in the *Other* condition, as compared to the *Solo* condition: (1) memorization performance was significantly higher (Exp. 1a) and (2) the amplitude of MMN responses was significantly greater (Exp. 1b). These results indicate the presence of an enhancement of both high- and low-level perceptual learning processes in case of the physical co-presence of a confederate (Fig. 2). In Experiment 2 (replicating sample), we replicated the presence of a significant difference between *Other* and *Solo* conditions, both at a behavioural and EEG level. Surprisingly, in the *Virtual* condition, memorization performance was significantly lower (Exp. 2a) and MMN responses were significantly reduced (Exp. 2b), as compared to the *Other* condition. No difference was found between the *Virtual* and the *Solo* condition (Exp. 2a–b) (Fig. 3). That is, coherently with previous studies^5^, the presence of a virtual confederate does not improve implicit and explicit learning as compared to physical presence.

Furthermore, in Exp. 1b and 2b, the correlation between the EEG signal and the Bayesian Surprise index, showed a significant (Figs. 2d and 3d) negative peak within the time window including the MMN (≈ 210 ms post-onset), thus confirming that MMN actually reflects implicit learning mechanisms.

Overall, our results demonstrate that, even in the absence of a joint action^18,59,60^, physical co-presence is sufficient to enhance both higher-level memory encoding and lower-level perceptual learning, expressed by a distinctive electrophysiological pattern, i.e., the significant enhancement of MMN waveforms. Such effects are generally interpreted as a consequence of the fact that shared attention on a specific sensory event makes it more valuable as compared to when the same event is not co-attended^2^. The present results demonstrate that this sensory up-weighting impacts not only on higher level memory encoding^15,61^ but also on low-level perceptual learning. More specifically, as shown by the significant correlation with the Bayesian Surprise index (which quantifies the informational value conveyed by each stimulus), the amplitude of MMN precisely indexes the sensory information weighting according to its estimated relevance^62,63^. Therefore, the significantly greater amplitudes of MMN responses in the *Other vs. Solo* condition may be interpreted as the result of a sensory up-weighting driven by physical co-presence. At a neurophysiological level, it has been suggested that this top-down control of sensory weighting may be implemented through the disinhibition of the post-synaptic gain of pyramidal cells, which are tuned to the attended sensory modality^63,64^.

In Exp. 2b the virtual co-presence of others did not enhance explicit memory encoding and perceptual learning as compared to physical presence, suggesting that attentional mechanisms acted as if subjects were alone. Previous research suggests that the effect of shared attention on memory can be reduced by psychological distance^5,6,23^, which operationalizes how “close” a partner is in time, space and relational dimensions^2^. Accordingly, as it has been suggested for previous similar results^5^, partners connected via videocall might have been perceived more distant psychologically, or relationally, and therefore failed to activate the shared-attention mechanisms responsible for memory enhancement. More explicitly, sitting together in the same room might be that sort of shared subjective experience that is able to modulate intergroup relations and psychological closeness^65^.

It might be argued that differences in learning outcomes across conditions, rather than being driven by the specific enhancement of perceptual learning dynamics, are related to changes in the general arousal level. In other words, the state of physiological reactivity of the subject might be simply heightened by the physical presence of the confederate. Importantly, general arousal-driven effects should equally affect responses to *Standard* and *Deviant* stimuli, rather than be solely focused on change detection responses. However, our control analysis performed on arousal-related components of the evoked potentials (such as N1) elicited by the responses to *Standard* sounds revealed no significant difference across conditions (see SI). Instead, significant modulations were focused on the responses to *Deviant* sounds. Crucially, this finding suggests the presence of a neural mechanism, specifically directed to optimize the processing of novel information.

The present results contribute to cumulative theoretical knowledge on social cognition, suggesting that during physical but not virtual presence of conspecifics, more neural resources are selectively devoted to surprising sensory states, thus amplifying mismatch responses. This neural up-weighting of sensory information may represent the underlying mechanism of *shared attention* effects, allowing individuals to best attune to relevant, shared information and groups to consolidate common knowledge^18^. The present results, if confirmed, might be of relevance in teaching and rehabilitation research. Future studies should be specifically directed to investigate the role of psychological/relational, contextual and technological variables that modulate perceived psychological distancing in physical and virtual settings, in order to maximize the learning advantages brought by shared attention mechanisms.

## Supplementary Information

Supplementary Information.
