# Supplementary material for: Physical but not virtual presence of others potentiates implicit and explicit learning
Source: Sci Rep. 2022 Dec 8;12:21205. doi: 10.1038/s41598-022-25273-4 (PMC9732282; doi:10.1038/s41598-022-25273-4)
Supplement: Supplementary file 1 — Supplementary Information. [file 41598_2022_25273_MOESM1_ESM.pdf]

Physical but not virtual presence of others potentiates implicit and explicit learning

Pietro Sarasso1, Irene Ronga1*, Elena Del Fante1, Paolo Barbieri1, Irene Lozzi1, Nicola Rosaia2, Alessandro Cicerale1, Marco Neppi-Modona1, Katiuscia Sacco1

1BIP (BraIn Plasticity and behaviour changes) Research Group, Department of Psychology, University of Turin, Italy

2 Department of Economics, Harvard University, Cambridge MA, USA

Supplementary Information

Bayesian Surprise

We compute the Bayesian surprise for each trial under a Beta-Binomial model of Bayesian learning of the stimulus probabilities (Ostwald et al., 2012). The model assumes that the brain implements a sequential learning procedure starting from an uninformative prior, updates this prior according to the subsequent observations, and computes Bayesian surprise as the Kullback-Leibler divergence between prior and posterior (Ostwald et al., 2012). Following Otswald et al. (2012), we use a variant of this model that assumes an exponential forgetting of stimuli which are observed in the distant past.

Formally, we assume that the probability of observing a low ($x = 0$) or high ($x = 1$) intensity stimulus at a given trial is described by a Bernoulli distribution with parameter $\mu \in [0, 1]$:

$$\Pr(x|\mu) = \mu^x (1-\mu)^{1-x}, \, x \in \{0, 1\}.$$

The true value of μ is unknown by the subject, and the initial uncertainty is modelled by means of an informative prior of type Beta, whose density function is uniform on the unit interval:

$$f(\mu) = 1, \, \forall \mu \in [0, 1].$$

On each trial, the prior is sequentially updated according to the observed data likelihood to form a posterior distribution over μ. After N trials, let $\boldsymbol{x}^N$ denote the sequence of observed stimuli

$$\boldsymbol{x}^N = \begin{bmatrix} x_1 \\ \vdots \\ x_N \end{bmatrix}$$

where $x_i \in \{0,1\}$ for each $i = 1, ..., N$. Under standard Bayesian learning, the posterior over μ is computed as follows. The probability of observing a $\boldsymbol{x}^N$ when the true parameter is μ is:

$$\Pr\left(\boldsymbol{x}^N|\mu\right) = \mu^{n_N}\left(1-\mu\right)^{m_N}.$$

Bayes rule implies that the posterior over μ is a Beta distribution with density

$$f\left(\mu|\boldsymbol{x}^N\right) = \frac{\Pr\left(\boldsymbol{x}^N|\mu\right) f\left(\mu\right)}{\int_0^1 \Pr\left(\boldsymbol{x}^N|\nu\right) f\left(\nu\right) \mathrm{d}\nu} = \frac{\mu^{n_N}\left(1-\mu\right)^{m_N}}{B\left(1+n_N, 1+m_N\right)}$$

where $n_N = |\{i : x_i = 1\}|$ is the nuber of high-intensity stimuli, $m_N = N - n_N$ is the number of low-intensity stimuli and B is the Beta function (ADD REF).

In order to account for a forgetting dynamics, instead of using the accumulative stimulus counts n_N and m_N in previous formula, the model employed here weights past observations according to an exponential function. Define the weighted stimulus counts n_N^τ and m_N^τ by

$$n_N^\tau := \sum_{i=1}^{N} \exp\left(-\frac{1}{\tau}\left(N-i\right)\right) x_i$$

and

$$m_N^\tau := \sum_{i=1}^{N} \exp\left(-\tau\left(N-i\right)\right)\left(1-x_i\right).$$

$\tau \geq 0$ is a parameter governing the forgetting dynamics: for $\tau = 0$ we have $n_N^\tau, m_N^\tau = n_N, m_N$, whereas increasing the value of τ implies that past observations are weighted less and less. This results in a posterior density given by

$$f^\tau\left(\mu|\boldsymbol{x}^N\right) = \frac{\mu^{n_N^\tau}\left(1-\mu\right)^{m_N^\tau}}{B\left(1+n_N^\tau, 1+m_N^\tau\right)}.$$

Finally, the model quantifies the degree of learning as the Bayesian surprise, or Kullback–Leibler divergence, between the prior and posterior distribution over μ after a given trial. Let $\boldsymbol{x}$ denote the full sample of observed stimuli. The Bayesian surprise after the N-th trial is given by:

$$\begin{aligned}\text{surprise}_N^\tau\left(\boldsymbol{x}\right) &\equiv KL\left(f^\tau\left(\cdot|\boldsymbol{x}^N\right)|f^\tau\left(\mu|\boldsymbol{x}^{N-1}\right)\right)\\ &\equiv \int_0^1 f^\tau\left(\mu|\boldsymbol{x}^{N-1}\right)\log\frac{f^\tau\left(\cdot|\boldsymbol{x}^{N-1}\right)}{f^\tau\left(\mu|\boldsymbol{x}^N\right)}\mathrm{d}\mu.\end{aligned}$$

Due to the use of conjugate priors, the Kullback–Leibler divergence can be evaluated analytically, which significantly simplifies the computation. [1]

[1] In our particular case, it can be shown that

$$\begin{aligned}\text{surprise}_N^\tau\left(\boldsymbol{x}\right) = &\log\frac{B\left(1+n_N^\tau, 1+m_N^\tau\right)}{B\left(1+n_{N-1}^\tau, 1+m_{N-1}^\tau\right)} + \left(n_N^\tau - n_{N-1}^\tau + m_N^\tau - m_{N-1}^\tau\right)\psi\left(2+n_{N-1}^\tau+m_{N-1}^\tau\right)\\ &-\left(n_N^\tau - n_{N-1}^\tau\right)\psi\left(1+n_{N-1}^\tau\right) - \left(m_N^\tau - m_{N-1}^\tau\right)\psi\left(1+m_{N-1}^\tau\right)\end{aligned}$$

where B is the Beta function introduced above and ψ is the Digamma function (ADD REF).

Extended Results

Experiment 1

Spatial Memory Task. The effect of physical co-presence was evaluated by comparing memorization accuracies in the Solo scenario (mean= 34.62 (72.125%); SD= ± 8.197) with accuracies from the Other Scenario (mean= 39.17 (81.25%); SD= ± 3,729). A paired sample t-test has been used for this purpose. Significant improvements were observed in memorization performaces corresponding to the Other scenario compared to the Solo scenario ($t17= -2.321$; $p= 0.0329$, $dz = 0.585$; Figure 1a).

Trial-by-trial correlation with Bayesian surprise. The correlation analysis between single trial amplitudes and Bayesian Surprise indicated that r values peaked over frontal electrodes (Fz) at similar latencies for Other and Solo scenarios (Figure1d). At Fz, r values corresponding to the Other and Solo both significantly differed from the costant 0 in three significant clusters which survived cluster correction, corresponding to the latencies of the MMN, the N2-P3a complex and the N4 components of the auditory evoked potential. In the following lines we report the latency of significant clusters at Fz for the Other scenario (parentheses indicate p values and t values at peak latencies): 130-244 ms ($p < 0.001$; $t = -6.39$); 262-375 ms ($p<0.001$; $t=5.675$); 499-497 ms ($p < 0.001$; $t= -6.43$). In the following lines we report the latency of significant clusters at Fz for the Solo scenario: 143-240 ms ($p<0.001$; $t=-6.102$); 265-383 ms ($p < 0.001$; $t= 6.043$); 418-551 ms ($p<0.001$; $t=-4.4$). These results confirmed our prediction, indicating that MMN indexes Bayesian perceptual learning in our study. Furthermore, and in accordance with data from the literature, we also found a correlation between model updating following surprising stimuli (i.e. sensory surprise) and the P3 and N400 components (Bennett et al., 2015; Kolossa et al., 2015).

MMN Results. ERPs elicited by the Other and Solo scenarios on Fz (where MMN peak amplitudes are registered) are reported in Figure 1b. Grand-average waveforms were comparable with previous studies on auditory frequency processing (Garrido et al., 2016; Sams et al., 1985). For both Other and Solo scenarios, the MMN differential wave obtained by subtracting Standard from Deviant average response, showed a negative peak over frontocentral at approximately 210 ms post-onset, coherently with previous findings (Figure 1c; Sams et al., 1985). Crucially, the point-by-point t-test performed on MMN differential waves (Other vs. Solo) revealed two significant time-clusters surviving cluster correction. The first centro-parietal cluster spanned across C3, Cp5, Cp1 and P7 electrodes at 193-215ms post-onset (Figure 1c), which corresponds to the MMN peak latency. This result confirmed that, as expected, MMN waveforms were significantly larger in the Other scenario compared to the Solo scenario. The scalp location of the effect of scenario on MMN differential waves is coherent with previous studies that localized the source of MMN responses to frequency deviance in the superior temporal gyrus (Jääskeläinen et al.,

2004; Molholm et al., 2005). Müller et al. (2002) PET study showed that

when contrasting frequency deviant and frequency standard sounds the largest magnitude of activation to frequency deviant sounds emerged in the lower part of the left posterior superior temporal gyrus. The second cluster surviving cluster correction is centred over frontal electrodes (Fp1, Fpz, Fp2, Fz, F4, Fc5) at 290-317ms post-onset. As it can be visualised in Figure1b this is possibly due to a more negative rebound of the P2 component following the presentation of Standard trials in the Solo scenario (following a larger P2 component in the Other scenario).

Experiment 2

Behavioral Memory Task. Mean accuracies (Figure2a) peaked in the Other scenario (mean=38.111 (79.397%); SD= ±6.406) and were reduced in the Solo scenario (mean = 33.722 (70.254%); SD= ± 9.473) and in the and Virtual scenario (scores mean = 30.611 (63.772%); SD= ± 10.393). In order to further explore the effect of scenarios we performed a one-way repeated-measure ANOVA with memorization accuracies as dependent variable and one within-subject factor: "Scenarios" (three levels: Solo, Other, Virtual). The one-way ANOVA on memory performance revealed a main effect of Scenario (F15 =7.157; p=0.003, η2=0.296).
The effect of physical and virtual co-presence was further evaluated by comparing memorization accuracies corresponding to the three different scenarios by means of three two-tailed paired sample t-tests (Other vs. Solo; Other vs. Virtual; Virtual vs. Solo). Significant enhancements in memorization performances were observed in the Other scenario compared to the Solo scenario (t17= 2.587; p= 0.019, dz = 0.731) and in the Other scenario compared to Virtual scenario (t17=4.071; p<0.001, dz = 1.061). Memorization performances in the Virtual and Solo scenarios were not significantly different (t17= 1.310; p=0.207; dz =0.312).
Trial-by-trial correlation with Bayesian surprise. As in Experiment 1, the trial-by-trial correlation analysis between single trial amplitudes and Bayesian Surprise indicated that r values peaked over fronto-central electrodes at three different latencies, similarly for the Other, Solo and Virtual scenarios (Figure 2d). Significant clusters corresponded to the latencies of the MMN, the N2-P3a complex and the N4 components of the auditory evoked potential. At Fz, results from the trial-by- trial correlation analysis performed on EEG responses following the presentation of the three scenarios, Other, Solo and Virtual intervals evidenced three significant clusters (i.e. r values significantly differed from the constant 0). For the Other scenario: 59-249 ms ($p < 0.001$; t= - 9.275); 264-347 ms ($p < 0.001$; t=4.88); 405-522 ms ($p < 0.001$; t=-5.84). For the Solo scenario: 129-247 ms ($p < 0.001$; t= -6.713); 265-362 ms ($p < 0.001$; t=5.659); 407-497 ms ($p < 0.001$; t=-5.788). For the Virtual scenario: 129-236 ms ($p < 0.001$; t= -5.72); 257-336 ms ($p < 0.001$; t=8.525); 419-555 ms ($p < 0.001$; t=-4.185). These results confirmed our prediction, indicating that MMN indexes Bayesian perceptual learning in our study. Furthermore, and in accordance with data from the literature, we also found a correlation between model updating

following surprising stimuli and the P3 and N400 components (Bennett et al., 2015; Kolossa et al., 2015).

MMN Results. ERPs elicited during the Other, Solo and Virtual scenarios intervals over Fz are reported in Figure 2b. Grand-average waveforms were similar to Experiment 1 and comparable with previous studies on auditory frequency processing (Sams et al., 1985). For Other, Solo and Virtual intervals, MMN waveforms showed a negative peak over fronto-central electrodes at approximately 220 ms post-onset (Figure 2c). The point-by-point t-test comparing MMN waveforms registered over Fz during the Other and Solo scenarios (Other vs Solo) revealed two early significant clusters corresponding to the N1 and MMN latencies, and a later significant frontal cluster at approximately 700ms post onset. The first smaller cluster, corresponding to the N1 negative peak spanned across frontal electrodes (Fp1, Fpz, F7, F3, Fc5) at 110-177ms post-onset. The second cluster including the time interval between 195 and 223 ms extending over a broad set of frontal, central and parietal electrodes (Fp1, Fpz, Fp2, F7, F3, Fz, FC5, FC1, FC2, FCz, T7, C3, Cz, CP1,CP2, CP6, P3, Pz, P4, Oz, O2 and POz) corresponded to the MMN peak latency. The point by point t-test comparing MMN registered during the Other and Virtual scenarios revealed a single significant cluster including the time interval between 237 and 278 ms post-onset, corresponding to the descending portion of the MMN negative peak. The significant cluster spanned across frontal channels. More specifically the results were significant over: FP1, Fpz, F3, Fz, F4, F8.
The t-test comparing Solo and Virtual scenarios (Solo vs. Virtual) was not statistically significant. Overall, as expected, MMN waveforms were significantly larger in the Other scenario (Figure 2c).

References

[1] Bennett, D., Murawski, C., & Bode, S. (2015). Single-Trial Event-Related Potential Correlates of Belief Updating. ENeuro. https://doi.org/10.1523/ENEURO.0076-15.2015

[2] Garrido, M. I., Teng, C. L. J., Taylor, J. A., Rowe, E. G., & Mattingley, J. B. (2016). Surprise responses in the human brain demonstrate statistical learning under high concurrent cognitive demand. Npj Science of Learning, 1(1), 16006. https://doi.org/10.1038/npjscilearn.2016.6

[3] Jääskeläinen, I. P., Ahveninen, J., Bonmassar, G., Dale, A. M., Ilmoniemi, R. J., Levänen, S., Lin, F. H., May, P., Melcher, J., Stufflebeam, S., Tiitinen, H., & Belliveau, J. W. (2004). Human posterior auditory cortex gates novel sounds to consciousness. Proceedings of the National Academy of Sciences of the United States of America. https://doi.org/10.1073/pnas.0303760101

[4] Kolossa, A., Kopp, B., & Fingscheidt, T. (2015). A computational analysis of the neural bases of Bayesian inference. NeuroImage. https://doi.org/10.1016/j.neuroimage.2014.11.007

[5] Molholm, S., Martinez, A., Ritter, W., Javitt, D. C., & Foxe, J. J. (2005). The neural circuitry of pre-attentive auditory change-detection: An fMRI study of pitch and duration mismatch negativity generators. Cerebral Cortex. https://doi.org/10.1093/cercor/bhh155

[6] Müller, B. W., Jüptner, M., Jentzen, W., & Müller, S. P. (2002). Cortical activation to auditory mismatch elicited by frequency deviant and complex novel sounds: A PET study. NeuroImage. https://doi.org/10.1006/nimg.2002.1176

[7] Ostwald, D., Spitzer, B., Guggenmos, M., Schmidt, T. T., Kiebel, S. J., & Blankenburg, F. (2012). Evidence for neural encoding of Bayesian surprise in human somatosensation. NeuroImage, 62(1), 177–188. https://doi.org/10.1016/j.neuroimage.2012.04.050

[8] Sams, M., Paavilainen, P., Alho, K., & Näätänen, R. (1985). Auditory frequency discrimination and event-related potentials. Electroencephalography and Clinical Neurophysiology/ Evoked Potentials. https://doi.org/10.1016/0168-5597(85)90054-1
